# Development and validation of a scale for measuring cultural beliefs about psychotherapy patients in southern Chile

**DOI:** 10.1186/s41155-020-0140-5

**Published:** 2020-02-17

**Authors:** Natalia Salinas-Oñate, María José Baeza-Rivera, Manuel Ortiz, Héctor Betancourt

**Affiliations:** 1 Departamento de Psicología, Facultad de Educación y Humanidades, Universidad de La Frontera, Avenida Francisco Salazar 01145, Casilla 54-D, Temuco, Chile; 2 Facultad de Ciencias de la Salud, Departamento de Psicología, Universidad Católica de Temuco, Manuel Montt 56, Temuco, Chile; 3 Department of Psychology, Loma Linda University, Loma Linda, CA 92350 USA

**Keywords:** Culture, Culturally pertinent instruments, Beliefs about psychotherapy patients, Mental health stigma

## Abstract

**Purpose:**

Negative cultural beliefs about psychotherapy patients represent one of the barriers in the psychological help-seeking and treatment adherence. In Chile today, there is little research about specific beliefs towards this group, and therefore measuring them represents a challenge. The aim of the present study was to develop and validate an instrument to measure cultural beliefs about psychotherapy patients.

**Methods:**

A mixed method design conducted in four stages was implemented. First, 32 semi-structured interviews were carried out to identify beliefs about psychotherapy patients in southern Chile. Then, a scale of beliefs about psychotherapy patients (SBPP) was developed and piloted in an adult sample (*n* = 109). Subsequently, the factorial structure of the new scale was explored in patients of primary health centres in La Araucanía Region of Chile (*n* = 201). Finally, the validity of the construct was assessed in adults who were not undergoing psychotherapy (*n* = 361).

**Results:**

The results showed the existence of negative cultural beliefs about psychotherapy patients which were included in the construction of the SBPP. The scale had a bifactorial structure (*α*_transitory situations_ = 0.81 and *α*_stable characteristics_ = 0.79), consisting of 15 items with a Likert-type response format, and showed good indicators of validity and reliability on the samples in which were applied.

**Conclusions:**

The present study shows the importance of using mixed methods for the examination of socially shared beliefs by the cultural group under study, in order to construct instruments that are psychometrically robust and culturally pertinent.

## Introduction

In Chile today, mental disorders are the principal source of disease, representing 23.2% of disability-adjusted life years (DALY) (Ministerio de Salud, 2017). Furthermore, those who present mental health problems must not only struggle with their primary condition, but also experience the secondary impact of the mental health stigma (Huggett et al., 2018). This has been catalogued as a “second disease” (Finzen, 1996), acting as a barrier to psychological help-seeking (ten Have et al., 2010), and being defined as a process involving labelling, separation, loss of status, endorsement of the stereotype, prejudice and discrimination, in a context in which social power is exercised on detriment of the members of a group of people (Link & Phelan, 2001). This situation has led the World Health Organization (2013) to call for a change in the attitudes that perpetuates these negative beliefs and discrimination.

One of the elements of stigmatization consists in holding on negative beliefs about mental health patients, perceiving them as unpredictable, violent and dangerous (Angermeyer & Dietrich, 2006). Gradations of these negative beliefs exist, with a stronger stigma attached to people diagnosed with schizophrenia (Bengochea-Seco et al., 2018; Caqueo-Urízar, Boyer, Urzúa, & Williams, 2019) or substance abuse than those with depression or anxiety. It may be noted that beliefs about the latter group are quite different, where the negative evaluation would consist in considering them emotionally unstable, uninteresting for interpersonal relations, incompetent and unreliable (Ben-Porath, 2002). However, the most striking finding is that these beliefs are more accentuated towards individuals who seek professional help (e.g. psychotherapy), since they are seen as more unstable, needing external assistance to deal with their psychological problems (Ben-Porath, 2002). This explains the negative impact attached to the processes of seeking psychological help.

This is important because untreated mental problems may potentially affect social relations, productivity and academic success (Hunt & Eisenberg, 2010). These, in turn, are risk factors for social isolation, inadequate medical attention and poor employment opportunities (Corrigan & Watson, 2002). Further, the results of the meta-analysis carried out by Clement et al. (2015) indicate that although it is clear that stigmatization has a negative impact on help-seeking, this concept is still not well understood.

This finding coincides with the situation in Chile, where—despite the incidence of mental health problems—there is no clear evidence to date on shared beliefs about psychotherapy patients, neither the existence of emic elements (unique to this cultural group) nor the replication of beliefs that have been found in other cultural contexts (Huggett et al., 2018).

The lack of evidence is not surprising given that only 9.6% of all health scientific publications in Chile are related to mental health issues (Ministerio de Salud, 2017), which hinders being well informed about the mental health field in the local context. Thus, this is consistent with the lack of a mental health law, to provide support for the whole set of actions necessary to improve mental health and to protect mental health patients. On the other hand, considering that the cultural context responds to a particular ecosystem (Triandis & Suh, 2002) and that at macro levels (such as the public policy of a country), it is possible to visualize the impact of culture (Van de Vijver, Chasiotis, & Breugelmans, 2011), it is expected that these definitions end up feeding back members of that culture. Subsequently, the above situation added to one of the lowest investments in mental health (2.16%) contribute to a vicious circle that is not very encouraging, where the message sent to the population seems to be “mental health is not relevant”, “mental health problems do not need professional advice” and consequently “mental health is not a priority”. At the international level, Corrigan and Watson (2002) indicate that negative beliefs about people seeking help for mental health problems are shared by the majority of the populations of the USA and western Europe, including not only the misinformed members of the community, but also well-trained professionals from mental health disciplines (Lin et al., 2019). These stereotypes become endorsed leading to prejudice and finally discrimination. Thus, stigmatized individuals may avoid seeking help to reduce the public stigma and negative consequences that result from it. In recognition of this fact, several instruments have been created to evaluate these beliefs. A widely used scale is the Stigma Scale for Receiving Psychological Help (SSRPH) (Komiya, Good, & Sherrod, 2000), which through five items, assesses stigma related to receiving psychological help (e.g. “Seeing a psychologist for emotional or interpersonal problems is a sign of personal weakness or inadequacy”).

The presence of such beliefs appears to be less evident in Asian and African countries, although it is not clear whether this finding represents a cultural sphere which does not promote stigmatization or merely a lack of investigation in these societies (Corrigan & Watson, 2002).

In Chile, there is no evidence of cultural beliefs about psychotherapy patients. Still, variables that could be associated with have been studied, identifying both positive and negative cultural beliefs about psychotherapy. For instance, positive beliefs are that psychotherapy enables the patient to feel that he/she can manage his/her own life, and negative beliefs are that psychotherapy aggravates the patient’s problems (Salinas-Oñate, Ortiz, Baeza-Rivera, & Betancourt, 2017). Furthermore, local studies indicate some concerns associated with being a mental health patient, such as “fear of the diagnosis” and “what other people might think” (Vicente et al., 2005). Nevertheless, the challenge of characterizing these stigmatizing beliefs at the local level has yet to be addressed (Tapia, Castro, Poblete, & Soza, 2015).

To assume the existence of a certain stigma requires, in the first place, the identification of cultural beliefs associated with psychotherapy patients, which eventually would lead on to study other characteristic elements of this construct (e.g. social exclusion, prejudice and stereotyping). In this context, and considering the different gradations remarked above (Angermeyer & Dietrich, 2006), it would seem relevant to focus on beliefs about neurotic rather than psychotic patients, since Chile has one of the highest rates of depression in Latin America (17.2%), and this disease—together with anxiety disorders—is one of the top five causes of DALY among women (Ministerio de Salud, 2017).

Considering the importance of these negative beliefs, both in seeking help and in the wide range of possible consequences for people’s lives, they need to be identified in the cultural group on which this study focuses, since they need to be measured properly with robust psychometric instruments in order to demonstrate the role they play in seeking help and other phenomena of interest (Betancourt & Flynn, 2009).

In view of the above situation, the aim of the present study was to develop and validate an instrument to measure cultural beliefs about psychotherapy patients.

## Method

The design of this research was non-experimental, cross-sectional and multivariate, using the methodology described by Betancourt, Flynn, Riggs, and Garberoglio (2010) for the construction of culturally pertinent instruments and based on the proposal of Triandis and Suh (2002) for the study of subjective culture. This proposal emphasizes the importance of considering various types of methods, looking for a convergence of findings across them. In addition, it is suggested to study different kinds of samples (e.g. women, men, different age group and others), starting with open question, like “what comes to your mind when you say…”, considering that when people give the same response to the same stimuli, this indicates the presence of shared components (Triandis & Suh, 2002).

Considering the above, the bottom-up/top-down cultural research approach was used (Betancourt et al., 2010), carried out in four phases: (1) identification of socially shared beliefs about psychotherapy patients, (2) construction and piloting of the new scale, (3) evaluating the factorial structure of the scale on people referred to psychotherapy, and (4) providing evidence of the construct validity in a sample of emergent adults who were not undergoing psychotherapy.

All these phases were conducted under the approval of the appropriate ethics committee (phases I and II = La Frontera University Ethics Committee, phase III = La Araucanía Sur Ethics Committee, phase V = Universidad Católica de Temuco Ethics Committee).

Considering the sequential nature of the phases of the investigation, they will be shown separately below, and the methodological aspects and principal results of each will be described.

### Phase I: Identification of cultural beliefs

#### Participants

A non-probabilistic, intentioned sample of 32 people aged over 18 years was selected, including different sources of cultural variation, stratifying the sample in terms of health system used (public and private) which accounts for variability in socioeconomic status, sex, and ethnicity, including members of the Mapuche ethnic group, indigenous minorities and the largest minority population in the country (Instituto Nacional de Estadisticas; INE, 2015). Participants had an average age of 34.1 years (SD = 10.4 years) and were mostly women (59.3%), and about half (46.9%) identified themselves as Mapuche. In regards to educational level, great diversity was observed, with the highest frequency cumulated in the category of complete high school level (21.9%), complete technical studies, and undergraduate level (both 18.8%). The majority of the sample (75%) belongs to the middle socioeconomic level, which includes medium-low, medium and medium-high level. Approximately half of the sample (53.1%) preferably use the public health system, 28% report no personal or close experiences (friends or family) in psychotherapy treatment, and of the remaining percentage, 56.3% rate the experience as positive.

#### Instrument

The data collection technique used was the semi-structured individual interview, based on the protocol implemented by Betancourt et al. (2010); the topics were chosen to explore the subjects’ beliefs about the people who attended psychotherapy or psychological care (e.g. “What do you think about people undergoing psychotherapy?”, “What is the first thing that comes to mind when you think about someone who attend psychotherapy?”).

#### Data analysis

The qualitative interviews were digitally recorded and transcribed into Atlas.ti 7.5 (Hwang, 2008), a qualitative data analysis software program. Three expert judges using thematic content analysis procedures (May, Strauss, Coyle, & Hayward, 2014) coded the interviews. In each stage, the information was triangulated to avoid potential bias in the process and comply with the rigour of verifiability (Guba, 1989), with an experienced external investigator assessing the suitability, quality and coherence of the content analysis of the interviews.

#### Results

**Fig. 1:**
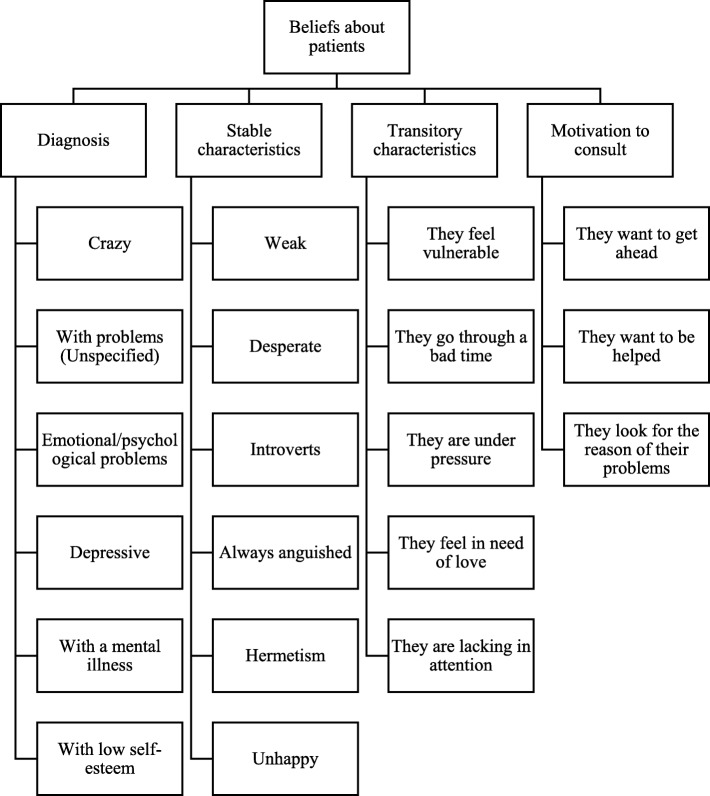
Thematic analysis of cultural beliefs about psychotherapy patients, bottom-up—phase I

The results of this stage show important cultural elements with respect to people who attend psychological treatment. As Fig. 1 shows, the beliefs reported most frequently refer to a condition or “diagnosis” described as “being crazy”, although this was reported as an opinion of other people and not one with which the interviewees identified. This large category (diagnosis) is followed by the stable characteristics of the patient which make him or her more likely to consult; this forms a list of qualities with negative connotations, the most frequent of which was “being weak”. This was followed by the category of transitory situations, which grouped codes referring to non-stable qualities of subjects and is associated with specific moments in which people need psychological attention (“they feel vulnerable”). Less frequent are codes referring to the reason why people consult (“they want to get over it”).

### Phase II: Development of instruments for measuring cultural variables identified in phase I

This phase aimed to develop an instrument for measuring cultural beliefs about psychotherapy (identified in the previous phase). To this end, items were created and passed through a content validation with expert judges. Subsequently, this first revised version of the instrument was piloted in a sample of adults of the general population, exploring the factor structure and reliability of the instrument. This process is briefly described below.

#### Development of items and content validation

The scale of beliefs about psychotherapy patients (SBPP) was developed based on the most frequently reported content codes. The original version consisted of 26 close-ended items, with a 5-point Likert-type response format (1 = totally disagree to 5 = totally agree).

These items were then subject to content validation by 12 expert judges (clinical psychologists) who assessed the coherence, sufficiency and clarity of the items. The degree of agreement of the scores given by each judge was estimated with Aiken’s V coefficient (Aiken, 1985); items with a value equal to or higher than 0.8 were accepted, those items whose value was between 0.70 and 0.79 were evaluated with caution, and those less than 0.70 were dropped out. On the other hand, the percentage of coherence of the item was calculated (percentage of judges who rated the item as “consistent”). Based on a combination of both criteria, items were selected and adapted if needed. Then, a second evaluation was carried out by the principal investigator (triangulated with another expert researcher), where new items were removed or added according to the redundancy or lack of items for a particular construct.

Considering the criteria mentioned above, six items were accepted without changes, 17 items were accepted incorporating wording changes, three items were rejected, and three new items were added, based on the suggestions of the judges. Finally, the scale was composed of 19 items.

#### Pilot testing of instruments

The new version of the scale was piloted with adults (*n* = 109) who attend public health services in La Araucanía Region. Participants had an average age of 38.3 years old (SD = 12.75), were mostly women (60%) of urban origin (74%) and identified themselves as non-Mapuche (75.5%). Most of the sample has an educational level between complete high school and complete technique studies (32.4 and 34.3%, respectively). Also, 53.7% have full-time work and belonged to medium-low (20.8%), medium (31.7%) and medium-high (32.7%) socioeconomic levels. On the other hand, 73% of the sample has never been under psychological treatment, and only 4.8% used medications for the management of anxious or depressive symptoms.

#### Data analysis

All the analyses were conducted with STATA version 14.1, adopting a nominal alpha of 0.05 (*p* < 0.05).

First, descriptive analyses were carried out to characterize the sample. Then the factorial structure of the scales was estimated with Exploratory Factorial Analysis (EFA). The factorization capacity of the matrix was evaluated with Kaiser Meyer-Olkin (KMO) sample-fitting measurements and Bartlett’s test of sphericity; KMO greater than or equal to 0.60 was considered adequate, while it was expected that the null hypothesis would be rejected by Bartlett’s test of sphericity (Pett, Lackey, & Sullivan, 2003).

The maximum likelihood component extraction method was used (ML) and oblimin rotation. The following criteria were used to make decisions on the best factorial solution: (a) eliminate factors with eigenvalues of less than one; (b) eliminate items whose factorial load was less than 0.30 and (c) eliminate factors with less than three items. The best factorial solutions were selected, considering the statistical results and previous findings documented in the literature. Then, the factors were named according to theoretical considerations.

Finally, Cronbach’s alpha was used to estimate the reliability of the scale (Cronbach, 1951).

#### Results

The results reflect a factorizable matrix; specifically, the KMO was 0.71, and Bartlett’s test of sphericity was significant (*χ*^2^ = 323.16, *p* < 0.001). From the exploratory factorial analysis, two independent factors were identified. The first one grouped five items and was called “stable characteristics” (*α* = 0.80), since the content of these items implied qualities which could not easily be modified (e.g. “psychotherapy patients have a weak character”). The second factor was called “transitory situations” (*α* = 0.65), given that the semantic content of the items denoted beliefs in which the patient was defined as a function of characteristics that are situational and, therefore, capable of change (e.g. “psychotherapy patients are disoriented”). The first factor explained 23.5% of the variance and the other 15.5%.

Considering that the second factor was only composed of three items, two new items were added, taking into account its theoretical suitability within the factor and according to the findings of the first phase of the study (e.g. “Psychotherapy patients are experiencing a crisis”) (more details in Table 1).

**Table 1:** Factorial structure, factorial loadings, explained variance and reliability of the SBPP scale—second version

Items (loadings)	Factor 1 “stable characteristics” (*α* = 0.80)	Factor 2 “transitory situations” (*α* = 0.65)
Have weak character	0.71	
Are unhappy	0.63	
Are nervous	0.63	
Are much troubled	0.58	
Are crazy	0.55	
Have a mental illness	0.49	
Need help to solve their problems	0.40	
Are depressed		0.73
Are going through a difficult time in their lives		0.61
Are disoriented		0.56
Variance explained (%)	23.7	15.5

Source: own elaboration

Footnote: Heading of all items = “psychotherapy patients:”

KMO = 0.71; Bartlett’s test of sphericity *χ*^*2*^ = 323.16, *p* < 0.00

Thus, based on the results of this procedure, the instrument was compounded by 12 items. It was further decided to add four items with a positive connotation (e.g. “people who attend psychotherapy are concerned about their personal well-being”) to avoid bias, although only the answers to the 12 original items were considered.

### Phase III: Evaluation of factorial structure of the scale

#### Participants

Two hundred one adult women who attended primary health care centres in La Araucanía Region were selected by using incidental non-probabilistic sampling. All the participants were referred to psychotherapy in the context of the Public Mental Health Programme. Those with a clinical diagnosis of severe depression who presented psychotic symptoms and who suffered a condition that limited their ability to answer the instruments (such as illiteracy, senile dementia, intellectual or visual disability, and cognitive deterioration) were excluded. It may be noted that the original sample consisted of 210 subjects, of whom only nine were men; the latter were therefore excluded from the final sample. This is consistent with most of the Chilean studies in mental health that report findings without incorporating men because more than 90% of the cases undergoing mental health treatment in the public system are women (Alvarado, Vega, Sanhueza, & Muñoz, 2005).

Participants were 44 years old (*M* = 43.7; SD = 16.4 years), the majority were of urban origin, and 28.4% belonged to the Mapuche ethnic group (further details in Table 2).

**Table 2:** Composition of the sample—phase III

	Descriptive
Age (SD)	43.7 (16.4)
Ethnicity (%)	
Non-Mapuche	71.6
Mapuche	28.4
Origin (%)	
Urban	94
Rural	6
Educational level (%)	
Incomplete primary	11.9
Incomplete high school	10.4
Complete high school	20.9
Incomplete technical studies	35.3
Complete technical studies	15.9
Undergraduates	5
Post grade	0.5
Socioeconomic status (%)	
Low	9.5
Middle low	43.8
Middle	26.9
Middle high	16.4
High	3.5
Previous psychological treatment (%)	
No	45.3
Yes	54.7
Medication (%)	
No	38.3
Yes	61.7

*SD* standard deviation, *%* percentages (frequencies)

#### Instrument

The SBPP (developed in phase II) was administered, together with a specially prepared socio-demographic questionnaire to characterize the sample.

#### Data collection procedure

All the participants were contacted in primary health care centres by the health professionals who referred them for psychotherapy, inviting them to participate and explaining briefly the aims of the investigation. Those who agreed to take part were given a closed envelope containing copies of the informed consent form, instructions on how to complete the survey and the instrument to be answered. Participants answered the instrument at home and then contacted the researchers by telephone to coordinate collection of the instruments. Participants were provided with the equivalent of five US dollars as compensation for their time.

#### Data analysis

Since the scale had variations concerning the number of items, it was decided to explore its factor structure with this sample, using the same statistical analyses described for phase II.

#### Results

The results reflect a factorizable matrix; specifically, the KMO was 0.79 and Bartlett’s test of sphericity was significant (*χ*^2^ = 612.01, *p* < 0.001). From the exploratory factorial analysis, two related factors were identified (*r* = 0.30; *p* < 0.001). Both factors were named the same as in EFA conducted in the previous phase. The first one grouped five items and was called “transitory situations” (e.g. “psychotherapy patients are undergoing a crisis”), and the second factor grouped six items and was called “stable characteristics” (e.g. “psychotherapy patients have a weak character”). The first factor explained 31% of the variance and the second 16%.

It should be noted that the item “psychotherapy patients need help to solve their problems” was dropped out from factor 1 since its factorial loading was smaller than 0.30.

The reliability of the first factor was 0.79 and of the second 0.69 (see Table 3).

**Table 3:** Factorial structure of SBPP (EFA)—phase III

Items (factor loading)	Primary health system patients (*n* = 201)	
	“Transitory situations” (*α* = 0.79)	“Stable characteristics” (*α* = 0.69)
Are undergoing a crisis	0.86	
Are confused	0.72	
Are depressed	0.65	
Are going through a difficult time in their lives	0.54	
Are disoriented	0.53	
Are much troubled		0.70
Have weak character		0.64
Are nervous		0.62
Are unhappy		0.43
Have a mental illness		0.37
Are crazy		0.32
Variance explained (%)	30.8	15.7

Source: own elaboration

Footnote: Heading of all items = “psychotherapy patients:”

KMO = 0.79; Bartlett’s test of sphericity *χ*^2^ = 612,66 *p* < 0.00

### Phase IV: Evidence of construct validity and reliability in emergent adults who were not undergoing psychotherapy

#### Participants

A non-probabilistic, intentioned sampling was used to select 361 undergraduate students (57% female) from universities in Temuco, Chile. The inclusion criteria were (a) being an undergraduate student and (b) age range 18 to 25 years old. The exclusion criteria were (a) presenting any visual or intellectual disability which would hinder them from answering the instruments properly; (b) being a psychology student, to avoid bias in the study subject; and (c) being currently undergoing psychotherapy. The mean age of the participants was 20.5 years (SD = 2.3 years). Eighty-four percent were from urban backgrounds and 29% declared themselves belonging to the Mapuche ethnic group. Almost half of the participants (43.9%) belong to pedagogy careers, followed by engineering (22.7%) and by ten careers with lower percentages, which together account for 22.5% (10.9% of participants do not report their career). Eighty-two percent rated themselves as middle socio-economic status (82.2%); 33% had previously undergone psychotherapy, and 2% were under anxiety or depression pharmacological treatment.

#### Instruments

The participants answered two instruments:

Sociodemographic questionnaire—a specially prepared questionnaire that measures variables that allowed characterizing the sample (e.g. age, ethnicity, origin, and socioeconomic status).

SBPP—instrument developed in the previous phases to measure cultural beliefs about psychotherapy patients. The final version consists of 15 items (including four positive fake items), with a Likert-type response format of five points (1 = totally disagree to 5 = totally agree), with two dimensions or factors: (1) *Transitory situations*, composed of five items that allude to beliefs about certain transient conditions through which psychotherapy patients would go through (e.g. “psychotherapy patients are undergoing a crisis” and “psychotherapy patients are going through a difficult time in their lives”) and (2) *Stable characteristics*, composed of six items that allude to beliefs about negative personal characteristics that psychotherapy patients would have (e.g. “psychotherapy patients have a weak character” and “psychotherapy patients are unhappy”) (see the final instrument in Additional file 1).

#### Data collection procedure

Participants were contacted in their classrooms by trained psychology students, who invited them to participate, explained the study aims, emphasizing that participation was voluntary and confidential. Those who agreed to participate signed an informed consent form and then completed the instrument; this procedure took about 30 min. Participants were not monetary compensated.

#### Data analysis

A confirmatory factorial analysis (CFA) was conducted to test the factorial structure obtained in the previous phase. Because multivariate normality was not assumed, the models were estimated with Satorra-Bentler correction, including Satorra-Bentler chi-squared test (SBx^2^), the comparative fit index (CFI > 0.95), Tucker-Lewis index (TLI > 0.90), root mean square error of approximation (RMSEA ≤ 0.06) and the standardized root mean square residual (SRMR < 0.05) (Ortiz & Fernández-Pera, 2018). In addition, the Lagrange multiplier test was applied in accordance with theoretical and empirical considerations. The internal consistency of the scale was estimated using Cronbach’s alpha.

#### Results

Initially, the model of two related factors proposed in phases II and III were tested. We compared model 1 with an alternative model (model 2) that add covariance error’s term as suggested by the Lagrange multiplier test and theoretical criteria discussed below. The goodness of fit indicators for both models is presented in Table 4.

**Table 4:** Goodness of fit indicators of tested models

Models	*χ*^2^ (gl)	*χ*^2^/gl	CFI	TLI	RMSEA (IC-90%)	SMRS	Δ*χ*^2^ (Δgl)
Model 1	238 (43)	5.53	0.84	0.79	0.11 (0.11–0.14)	0.09	–
Model 2	123.87 (38)	3.26	0.93	0.90	0.08 (0.07–0.10)	0.07	114,13* (5)

Model 1 = Two correlated factors without covariances between items’ errors; Model 2 = Two correlated factors with five covariances between items’ errors. * *p* < 0.001

When comparing models 1 and 2, there is a significant difference. Also, model 2 shows better goodness of fit indicators that model 1. Given these differences in addition to the theoretical relevance of the second model, this one was chosen.

**Fig. 2:**
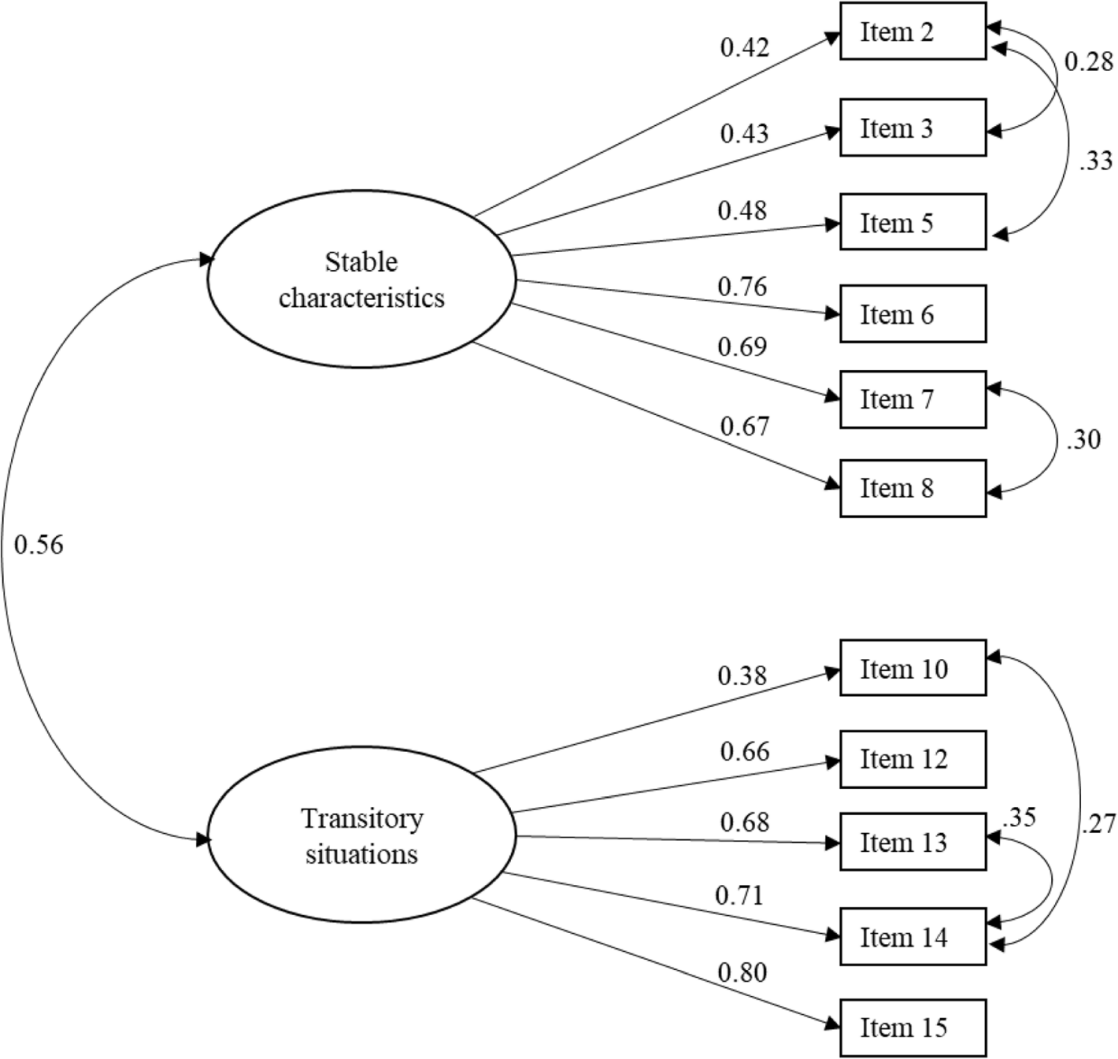
Confirmatory factor analysis of the SBPP—phase IV. CFI = 0.93, SB*χ*^2^ (38, *n* = 361) = 123.87, *p* = 0.00, RMSEA = 0.080 (00.07–0.10), TLI = 0.90, SRMR = 0.07

As Fig. 2 shows, all the factorial loadings were greater than 0.30, and the correlation between both factors was statistically significant (*r* = 0.56; *p* < 0.001).

Lagrange multiplier test suggested correlating some error terms. In “stable characteristics” factor, item 2 (“psychological care patients are crazy”) covariates with 3 (“are unhappy”) and 5 (“have a mental illness”), which could be showing the complexity of the cultural meaning of “being crazy” that is associated not only to have a mental health disorder, but also to have an unhappy life. Further, in “transitory situations” factor, item 14 (“psychotherapy patients are undergoing a crisis”) covariates with 13 (“are depressed”) and 10 (“are going through a difficult time in their lives”), which is concordant in theoretical terms, because these three situations share common characteristics, where a depressive episode is effectively interpreted as a critical and challenging moment.

The total internal consistency was 0.81. The reliability for factor 1 (*α*_transitory situations_ = 0.81) and factor 2 (*α*_stable characteristics_ = 0.79) was good, providing empirical evidence in favour of the instrument’s reliability.

## Discussion

The aim of this study was to develop and validate an instrument for measuring cultural beliefs about psychotherapy patients. It was addressed with a mixed design that considers four phases, enabling us to identify cultural beliefs associated with and subsequently to develop an instrument to measure them. Thus, we developed a scale that easily measures culturally shared beliefs about psychotherapy patients in our context.

Specifically, while we were developing the instrument, we identified the presence of cultural beliefs with negative connotations, grouped into two different but associated factors: the first one, called “transitory situations”, in which negative beliefs were not attributed directly to the patient but to the situations he/she lives; while the second one, named “stable characteristics”, refers to patients’ negative qualities. The first factor may be related to the evidence showing negative beliefs less accentuated towards patients diagnosed with anxiety or depression than towards psychiatric patients (Angermeyer & Dietrich, 2006). Nevertheless, due to the positive correlation between the two factors, although the former does not have such a strong load of stigmatizing contents, they are associated with the presence of stable beliefs that tend to stereotype psychotherapy patients as people with negative characteristics.

These dimensions appear through different samples under study and despite the process of scale refinement modifications. The dimensions mentioned above were the result of a reasonably congruent process. They were envisioned in four primary categories in phase I (diagnosis, stable characteristics, transitory situations, and motivation to consult). And then, from phase II onwards, they appear consistently as two dimensions, where the “diagnosis” (of phase I) category was theoretically included in the “stable characteristics” dimension and that the “motivation to consult” category was implicit in the dimension “transitory situations”, as well as in the creation of the items of positive connotation.

It should also be noted that we did not find the same contents reported in the international literature, where interpersonal performance is expressed in the negative characterization of patients (e.g. interpersonally uninteresting) (Ben-Porath, 2002). The present study, in contrast, shows negative beliefs associated with characteristics from the emotional sphere, such as being permanently anxious (“they are nervous”) or “being unhappy”, which perfectly could be the prelude to a poor social performance, nevertheless, which does not appear spontaneously in this context. A negative value is also attached to the presence of mental health problems (“have a mental illness”, “they are crazy”). The expression “having a weak character” is not limited to a single definition, and—based on the bottom-up phase—it may refer to (a) difficulty in taking decisions, (b) being easily influenced by others, (c) being emotionally labile and (d) having difficulty in dealing with own problems.

Although this scale was conceived to measure beliefs about psychotherapy patients, the findings obtained evidence that part of these beliefs is to assume that one of the main reasons for being in a psychotherapeutic process is to have a diagnosis of mental health, represented in the category “diagnosis” evidenced in phase I and—as a consequence—in several items of the “stable characteristics” dimension. In this regard, there are scales created in South America that measure beliefs about mental health patients, in particular Beliefs about Mental Illnesses Scale (BAMIS) (Maciel, Pereira, de Lima, & de Souzac, 2015), created from a successive phase methodology (similar to the one in this article) where representations about the causes of mental illness were associated with beliefs of diverse nature, for example, psychological, biological, and religious, among others. Notably, items belonging to the psychological nature of mental illnesses (for example, emotional instability or emotional problems, and excess of concern with life) are very similar to some items of the stable characteristics dimension of the present scale.

We also found covariances between items that belong to the same factor, that allow us to better understand the association between the contents addressed in those items and capture the complexity in which constructs are understood in each cultural group.

In view of the above, we corroborated the importance of caution in applying scales taken from other cultural contexts, and to consider the creation of new scales (e.g. Maciel et al., 2015), or adapting external scales transculturally (e.g. García, Barraza-Peña, Wlodarczyk, Alvear-Carrasco, & Reyes-Reyes, 2018), especially in order to measure cultural variables, since there is a risk of addressing contents which are not pertinent to the local context.

This study has strengths and limitations. To our knowledge, this is one of the first studies in Chile investigating beliefs about psychotherapy patients. Thus, we identified two correlated factors about these beliefs and created a valid and reliable measure that allows for an easy and fast measure, given its small number of items and simplicity of its content. Further, we used a mixed approach, culturally oriented, that is responsible for examining culturally relevant beliefs of this group with a diverse sample. The statistical analysis used allowed us to explore and then confirm a factorial structure consistent with the cultural meaning that psychotherapy patients have in our participants. The sample size is suitable, considering how challenging is the access to patients attending psychotherapy.

In line with the aforementioned, this procedure includes—to a great extent—recent suggestions for scale development process (Morgado, Meireles, Neves, Amaral, & Ferreira, 2018), where three basic steps were included: item generation, theoretical analysis and psychometric analysis. On the other hand, for content validity assessment, expert judges and target population judges (pilot sample) were considered. In addition, for construct validity, a combination of EFA with CFA was used, considering the “observations to variables” ratio of 15:1 or 20:1, respectively.

Nevertheless, our sample was composed only by women in the third phase. Although this is consistent with the Chilean profile of patients requesting psychotherapy, we suggest having caution when interpreting these results. Thus, women have more significant mental health issues than men, because of their multiple demands derived from the different roles they play, generating more considerable related distress and unhappiness (Behar, de la Barrera, & Michelotti, 2002), but also, they got more mental health disorders diagnosis from health care professionals (Cova, Valdivia, & Maganto, 2005). On the contrary, men experience higher social pressure for avoiding seeking psychotherapy because they must adhere to what it is expected to their gender (Pleck, Sonenstein, & Ku, 1994). Therefore, it is not surprising that our sample in the third phase was only female. This situation was intended to correct by having a mixed sample in the last stage.

Although the findings indicate the presence of negative beliefs about psychotherapy patients, our study did not include a behavioural measure of the actual use of it. Thus, we cannot prove whether these beliefs will predict the seeking of psychotherapy. Similar studies have included a measure of the behavioural intention to seek professional health care, which seems to be an easy and effective way to approach the actual behaviour.

Finally, we suggest that future studies could address the differential impact of transitory and stable beliefs on important processes as psychological help-seeking in the population, in order to better understand the impact of cultural variables in people’s health behaviours and consequently in their mental health.

## Conclusion

The present work illustrates the implementation of a mixed method (bottom-up top-down) and culturally oriented design for the construction of a scale to measure cultural beliefs about psychotherapy patients in southern Chile. These results support some of the findings of previous research conducted internationally, concerning negative beliefs about psychotherapy patients. However, findings evidence specific beliefs that are shared among this particular group, highlighting the need of using instruments that are capable of adequately measuring cultural constructs such as the one addressed in this study.

An accurate measurement of cultural beliefs about psychotherapy patients may lead researchers to a more comprehensive understanding of seeking and adhering to psychological care and contribute to more effective and culturally based interventions, particularly with culturally diverse and economically disadvantaged populations.

## Supplementary information

**Additional file 1.** Original SBPP (and English translation in italics).

## Data Availability

The datasets used and/or analyzed during the current study are available from the corresponding author on reasonable request.

## Abbreviations

BAMIS: Beliefs about mental illnesses scale
CFA: Confirmatory factorial analysis
CFI: Comparative fit index
DALY: Disability-adjusted life years
EFA: Exploratory factorial analysis
KMO: Kaiser Meyer-Olkin test
ML: Maximum likelihood estimation
RMSEA: Root mean square error of approximation
SBPP: Scale of beliefs about psychotherapy patients
SBx^2^: Satorra-Bentler chi-squared test
SRMR: Standardized root mean square residual
SSRPH: Stigma Scale for Receiving Psychological Help
TLI: Tucker-Lewis index
