# Supplementary material for: Development and validation of a scale for measuring cultural beliefs about psychotherapy patients in southern Chile
Source: Psicol Reflex Crit. 2020 Feb 17;33:2. doi: 10.1186/s41155-020-0140-5 (PMC7026340; doi:10.1186/s41155-020-0140-5)
Supplement: Supplementary file 1 — Additional file 1. Original SBPP (and English translation in italics). [file 41155_2020_140_MOESM1_ESM.docx]

**Appendix A** Original SBPP (and English translation in italics)

**Las siguientes afirmaciones muestran lo que algunas personas piensan o sienten en relación a las personas que buscan ayuda psicológica (pacientes).** Marca con un X el número que indica qué tan de ACUERDO o en DESACUERDO estás con cada una de las siguientes afirmaciones:

***The following statements show what some people think or feel about people seeking psychological care (patients).*** *Mark with an X the number that indicates how much you AGREE or DISAGREE with each of the following statements:*

| **Las personas que van al psicólogo:**  ***Psychotherapy patients:*** | | **Totally disagree** | | | **Totally agree** | | |
| --- | --- | --- | --- | --- | --- | --- | --- |
| 1. | se interesan por ser mejores personas*  *are interested in being better people* | ① | ② | ③ | | ④ | ⑤ |
| 2. | están locas  *are crazy* | ① | ② | ③ | | ④ | ⑤ |
| 3. | son infelices  *are unhappy* | ① | ② | ③ | | ④ | ⑤ |
| 4. | son más maduras que el resto de las personas*  *are more mature than the rest of the people* | ① | ② | ③ | | ④ | ⑤ |
| 5. | tienen una enfermedad mental  *have a mental illness* | ① | ② | ③ | | ④ | ⑤ |
| 6. | son nerviosas  *are nervous* | ① | ② | ③ | | ④ | ⑤ |
| 7. | son débiles de carácter  *have weak character* | ① | ② | ③ | | ④ | ⑤ |
| 8. | se aprobleman mucho  *are much troubled* | ① | ② | ③ | | ④ | ⑤ |
| 9. | se preocupan de su bienestar personal*  *care about their personal well-being* | ① | ② | ③ | | ④ | ⑤ |
| 10. | están pasando por un momento difícil en su vida  *are going through a difficult time in their lives* | ① | ② | ③ | | ④ | ⑤ |
| 11. | son capaces de asumir que tienen un problema*  *are able to assume that they have a problem* | ① | ② | ③ | | ④ | ⑤ |
| 12. | están desorientadas*  *are disoriented* | ① | ② | ③ | | ④ | ⑤ |
| 13. | están depresivas  *are depressed* | ① | ② | ③ | | ④ | ⑤ |
| 14. | están viviendo una crisis  *are undergoing a crisis* | ① | ② | ③ | | ④ | ⑤ |
| 15. | están confundidas  *are confused* | ① | ② | ③ | | ④ | ⑤ |

*= items with positive connotation
